# Retraction Note: Monitoring of production of blood components by attribute control chart under indeterminacy

**DOI:** 10.1038/s41598-022-15749-8

**Published:** 2022-07-04

**Authors:** Nasrullah Khan, Muhammad Aslam, P. Jeyadurga, S. Balamurali

**Affiliations:** 1 Department of Statistics and Computer Science, University of Veterinary and Animal Sciences, Jhang Campus, Lahore, 54000 Pakistan; 2 Department of Statistics, Faculty of Science, King Abdulaziz University, Jeddah, 21589 Saudi Arabia; 3 Department of Computer Applications, Kalasalingam Academy of Research and Education, Krishnankoil, TN 626126 India

Retraction of: *Scientific Reports* 10.1038/s41598-020-79851-5, published online 13 January 2021

The Editors have retracted this Article. Following publication, concerns were raised about the rationale for the approach presented and the underlying reasoning. A post-publication review of the authors' mathematical arguments revealed a lack of clarity in the terms presented and inferences that are not adequately justified. The Editors therefore no longer have confidence in the conclusions presented.

Nasrullah Khan and Muhammad Aslam do not agree to this retraction. P. Jeyadurga and S. Balamurali did not respond to correspondence relating to this retraction.

